# Epidemiological Challenges in Rare Bleeding Disorders: FVIII Inhibitor Incidence in Haemophilia A Patients—A Known Issue of Unknown Origin

**DOI:** 10.3390/ijerph18010225

**Published:** 2020-12-30

**Authors:** Christine Keipert, Ursula Drechsel-Bäuerle, Doris Oberle, Mirco Müller-Olling, Anneliese Hilger

**Affiliations:** 1 German Haemophilia Registry, Paul-Ehrlich-Institut, Federal Institute for Vaccines and Biomedicines, 63225 Langen, Germany; 2 Safety of Medicinal Products and Medical Devices, Paul-Ehrlich-Institut, Federal Institute for Vaccines and Biomedicines, 63225 Langen, Germany; Ursula.Drechsel-Baeuerle@pei.de (U.D.-B.); Doris.Oberle@pei.de (D.O.); 3 Hematology and Transfusion Medicine, Paul-Ehrlich-Institut, Federal Institute for Vaccines and Biomedicines, 63225 Langen, Germany; Mirco.Mueller-Olling@pei.de (M.M.-O.); anneliese.hilger@pei.de (A.H.)

**Keywords:** rare diseases, epidemiology, haemophilia A, inhibitor development

## Abstract

There is a broad range of factor products approved in Germany for haemophilia A treatment. Since the introduction of recombinant coagulation factor VIII (FVIII) products in the 1990s, there has been substantial debate whether there is a difference in inhibitor incidence between single FVIII products or product classes. Neither haemophilia registries nor clinical studies, including a randomised controlled clinical trial, provided a consistent and definite answer. The reasons were mainly related to methodological challenges in conducting controlled studies in a rare disease. In this analysis, the most relevant epidemiological challenges and main problems were examined, including study bias, potential overlap of individual studies and advanced development of therapy and methods in the course of time. Meta-analyses on two levels showed that therapies using recombinant products resulted in different event rates when compared to plasma-derived products. These results are accompanied by substantial study heterogeneity evidenced by Cochran’s Q tests. Only three studies have been identified that meet the standards of current clinical guidance. To finally resolve this ongoing and disputable safety issue of replacement therapy, collaboration among registry owners, academia and regulators must be fostered.

## 1. Introduction

**Figure 1:**
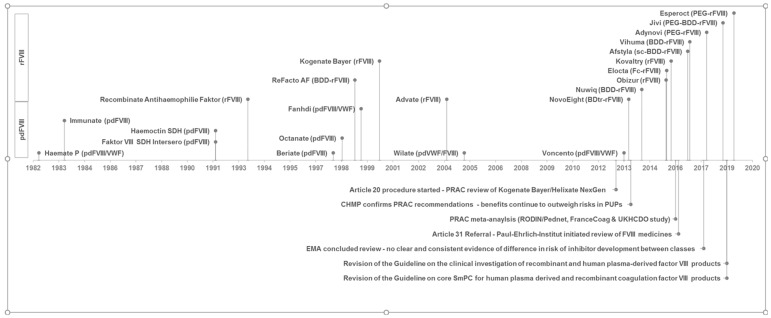
Time relationship between coagulation factor VIII (FVIII) product approval and triggered regulatory procedures for investigating the inhibitor incidence. License dates of human plasma-derived FVIII products (pdFVIII) and recombinant FVIII products (rFVIII) with marketing authorisation valid in Germany according to [18] are shown in the lower and upper area above the timeline. Qualitative product description is also given in brackets. VWF: von Willebrand factor, sc: single-chain, BDD/BDtr: B-domain deleted/truncated, PEG: PEGylated, Fc: Fc fusion protein.

Haemophilia A (HA) is a rare X-linked bleeding disorder caused by a mutation in the gene coding for coagulation factor VIII (FVIII). Annually about 250 newborns in Europe are diagnosed with the severe form of the disease, characterised by <1% residual FVIII activity. Those patients are in need of treatment with exogenous FVIII purified from blood or biotechnologically manufactured, referred to as plasma-derived FVIII (pdFVIII) or recombinant FVIII (rFVIII), respectively. The most serious and challenging side effect in patients with severe HA is the development of inhibitors against FVIII, which occurs in around 30% of the previously untreated patients (PUPs), primarily within the first 50 exposure days (EDs) [1]. There is a broad range of factor products approved in Germany for HA treatment (Figure 1) and since the introduction of rFVIII in the 1990s, there has been substantial debate whether there is a difference in inhibitor incidence between FVIII products [2,3]. In particular, the immunogenicity of second or third generation rFVIII products, von-Willebrand (vWF) content in FVIII products and rFVIII products modified for half-life extension gave rise to concern [4]. Neither haemophilia registries [5] nor clinical studies performed in the frame of marketing authorisation [6] nor a randomised controlled trial (RCT) [7] provided a definite answer. The reason was the lack of a sufficient number of patients with this rare disease to enable statistically relevant conclusions based on individual products as well as methodological problems.

Several meta-analyses or reviews have been conducted to elaborate on the role of different FVIII products for inhibitor incidence in PUPs [8,9,10] without conclusive findings for distinct products. Two regulatory reviews in the European Union under Article 20 of Regulation (EC) No. 726/2004 [11,12] and Article 31 of Directive 2001/83/EC [13,14] could not finally clarify whether there is a differential risk between product classes or individual recombinant products. Subsequently, the Guideline on the FVIII core SmPC was revised to include inhibitor development as a very common side effect in PUPs [15]. The time relationship between FVIII product approval and triggered regulatory procedures for investigating the inhibitor incidence is demonstrated in Figure 1.

Several factors led to significant changes in the European Medicines Agency (EMA) guidelines for FVIII products [16,17] and finally to the deletion of the obligation to perform clinical trials in PUPs for marketing authorisation purposes:

1. A sufficient number of factor products had already been approved for which risks and side effects in the treatment of PUPs were known.
2. It was foreseeable that many products would be seeking marketing authorisation at the same time so that due to the limited number of PUPs, this competitive situation would prevent studies being able to recruit patients in a reasonable time.
3. The studies and meta-analyses already conducted showed that the results of these studies are difficult to compare with each other.

We performed a systematic review of interventional and non-interventional studies and meta-analyses addressing inhibitor development in PUPs with severe HA. We hypothesise that, despite the number of patients involved in the respective studies being sufficient for statistical analyses, the risk of study bias and unmeasured confounding affect the meaningfulness of the calculation of inhibitor incidences. Therefore, we calculated combined inhibitor rates for plasma-derived and recombinant products for a carefully selected number of studies.

## 2. Materials and Methods

### 2.1. Study Selection

A protocol for the review was pre-specified and registered on PROSPERO. The search was designed to be as sensitive as possible. Both published and unpublished reports were considered. All types of studies were eligible. Studies were identified by searching electronic databases (MEDLINE, the Cochrane Controlled Trials Register, EMBASE, BIOSIS) from 1946 to 30.05.2018. The following terms were used for the search: factor viii, hemophilia a, previously untreated, minimally treated (see search example in Supplement File S1). Articles from scanning reference lists of papers and reviews were also included. No limits were applied for language, and non-English papers have been translated where possible. Two authors conducted title and abstract screening independently. Those references that were not considered relevant by both reviewers were excluded from the review. Following criteria were applied:

(1) Is this a primary study?
(2) Was a FVIII product administered to a PUP or MTP (minimally treated patient)?
(3) Did the study examine the risk of Factor VIII inhibitor development?

Full texts for the remaining references were obtained, and eligibility was checked by two individuals according to the criteria mentioned above.

### 2.2. Data Collection

Data extraction tables and forms were developed to standardise the information extracted from each of the eligible studies. The following data from each included report were extracted: author and publication year, study location, recruitment period, planned period of patient follow-up, study design (controlled/uncontrolled, interventional/non-interventional, prospective/retrospective), FVIII product administered (type, subtype and brand name, vWF content), number of PUPs and MTPs included, definition of severity, number of PUPs/MTPs with a defined severity, number of inhibitors, number of PUPs/MTPs with inhibitor development, number of patients with high titre (>5 BU)/low-titre/transient inhibitor, frequency of inhibitor testing specified, considered risk factors (race, genetics, family history, intensity of treatment, age), statistics (descriptive, hazard rate, Kaplan–Meier, other). Parameters were collected according to the Guideline on the clinical investigation of recombinant and human plasma-derived factor VIII products (ClinGL, [17]) (≥50 PUPs included (MTPs not counted), follow-up ≥ 50 EDs, severe haemophilia defined as <1% FVIII:C, (the Nijmegen modification of) Bethesda method used, inhibitor testing frequency defined and according to the ClinGL). Two independent researchers crosschecked extracted data.

### 2.3. Data Analysis

Biases reflect inadequacies in the design or conduct of a study and may affect the validity of the findings. Biases have to be assessed and accounted for in studies. Therefore, for individual studies, a “Risk of bias” table for each interventional and observational study according to reference [19] was completed. The reviewers were guided by signaling questions and required to make a judgement on the level of each type of bias found within the study.

Risks of bias due to confounding, selection, information, reporting, departure from intervention, and missing data have been assessed as low, moderate or serious. The authors identified the following relevant confounders that may introduce a bias if these confounders were not considered for the analysis by stratification and adjustment: age at first FVIII exposure, intensity of FVIII treatment, discrimination of treatment: prophylaxis or on demand, risk factors for inhibitors: ethnicity, severity, family history and genetics.

Risk of overlap of individual studies: The risk of overlap of individual studies was evaluated depending on centres respectively countries where the trial was performed, the recruitment period and the investigators of the study, e.g., if there were several publications with the same product, for all studies with a comparable number of patients, published over several years from the same investigators in the same countries, it could not be excluded that these publications are based on the same cohort of patients.

### 2.4. Statistical Analysis

In addition to a narrative synthesis of the study data, two meta-analyses were performed. Eligible for inclusion in the first meta-analysis were studies in PUPSs with severe disease (FVIII < 1%) for whom data on the development of any titre inhibitors after administration of FVIII products were provided, whereas in the second meta-analysis only studies were eligible that fulfilled all parameters specified in the ClinGL.

For each eligible study and product investigated, event rates (number of PUPs with any or high titre inhibitors/number of PUPs recruited) with corresponding 95% confidence intervals (CI) were calculated. Combined event rates were estimated by FVIII product type (plasma-derived; recombinant 1, generation full length; recombinant 2, generation full-length; recombinant 2, generation B domain deleted; recombinant 3, generation full-length; recombinant 3, generation B domain deleted; recombinant not further specified). In addition, an overall combined event rate was computed. A random-effects model was used because of the anticipated clinical heterogeneity of the studies.

Heterogeneity was assessed using the Q-test, which informs about the presence versus the absence of heterogeneity and the I^2^ statistic quantifying the extent of heterogeneity [20]. In addition, as an estimate of the between-study variance in a random-effects meta-analysis, tau-squared (τ^2^) was computed.

The meta-analyses were performed using the software Comprehensive Meta-Analysis, version 2.2.064 (Biostat, Englewood, NJ, USA).

## 3. Results

### 3.1. Study Selection

**Figure 2:**
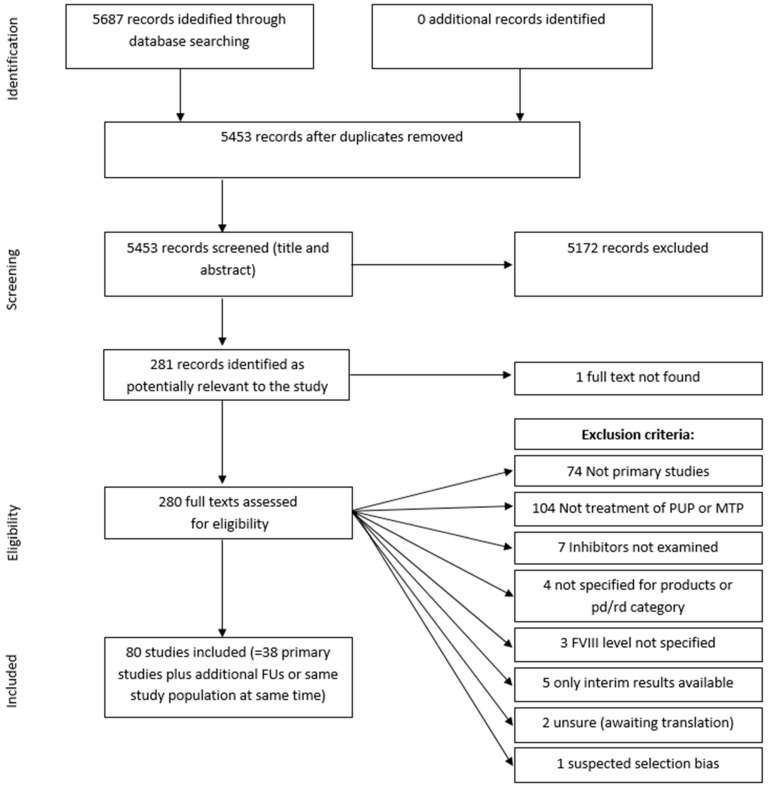
Flow of information from screening and inclusion process.

A total of 5687 records were identified through the electronic literature search. After the exclusion of duplicates, the title and abstract of 5453 records were screened, and 281 records were identified as potentially relevant for the study. The further screening and inclusion process is shown in Figure 2. The 80 publications identified as eligible in the final assessment were compared, and those covering the same patient population (e.g., the same study described in yearly updated publications) summarised. This finally led to 38 primary publications.

### 3.2. Relevant Publications According to the Parameters Demanded in the ClinGL

**Figure 3:**
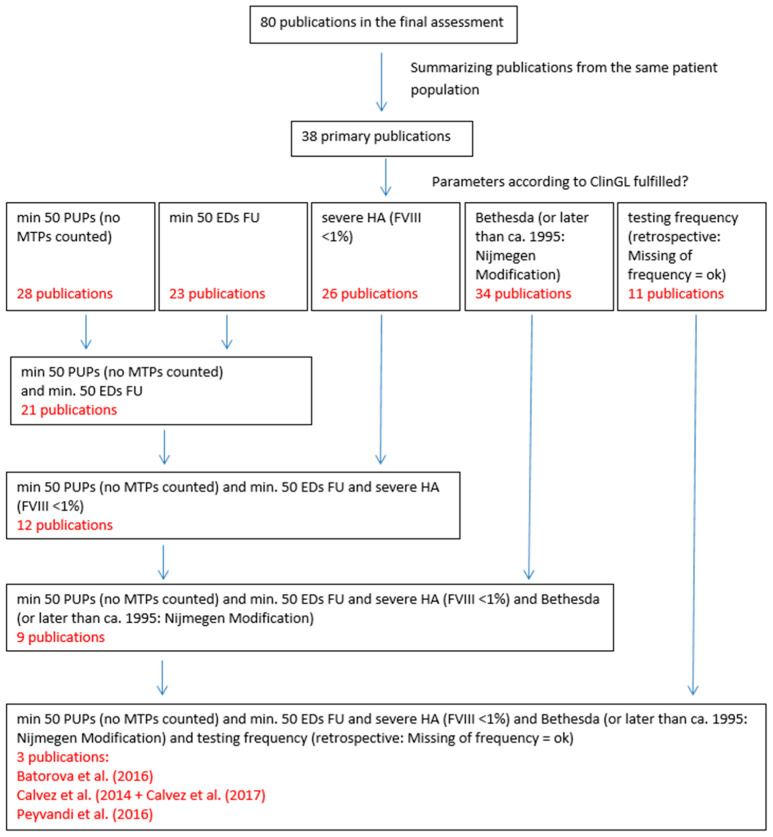
Relevant publications according to the parameters demanded in the ClinGL. A table of these 80 publications and the assessment process can be found in the Supplement to this paper (File S2).

Taking the parameters according to the ClinGL [17] into account, the following numbers were identified: 28 publications described a population of a minimum of 50 PUPs, 23 publications had a follow-up of 50 EDs or more, in 26 publications, severe HA was defined as FVIII:C < 1%, 34 publications reported that the Bethesda assay (or after 1995 the Nijmegen modification of this assay) was used, and in 11 publications a schedule for testing for inhibitors was defined prospectively. The combination of these conditions resulted in three publications that were in accordance with the ClinGL, which were, therefore, considered the most appropriate comparison groups. (Figure 3) [7,21,22].

### 3.3. Risk of Bias in Individual Studies

The completion of a “Risk of bias” table for each study according to Sterne et al. [19] led to the following result: Overall, the risk of bias for the 38 primary publications was rated low to serious. However, for some studies, little or no information was available regarding bias (Table 1). The quality of studies was reviewed in relation to the presence of potential confounders that could hamper the interpretation of the results. With respect to bias by confounding, the following factors were considered: discrimination of treatment: prophylaxis or on demand (n = 22 studies), FVIII gene mutation testing (n = 18 studies), severity of HA (n = 38 studies), human leukocyte antigen (HLA) genotype testing (n = 1 study), ethnicity (n = 28 studies), genetics (n = 19 studies), family history (n = 21 studies), intensity of treatment (n = 28 studies) and age (n = 38 studies). In addition, whether an adjustment for potential confounders with corresponding regression analyses was performed in the studies was checked, with the result that Cox proportional hazard models with time-dependent variables were applied to 11 studies. An overview of the underlying rating process can be found in the Supplemental Data (File S3). Of note, interventional studies might also be subject to a selection bias if PUPs were predominantly included. This might hinder the inclusion of patients who were born in families where HA was previously unknown and will only be detected in an emergency (around 50% of PUPs) or patients with peak treatment as the first treatment.

### 3.4. Potential for Overlap of Individual Studies

**Figure 4:**
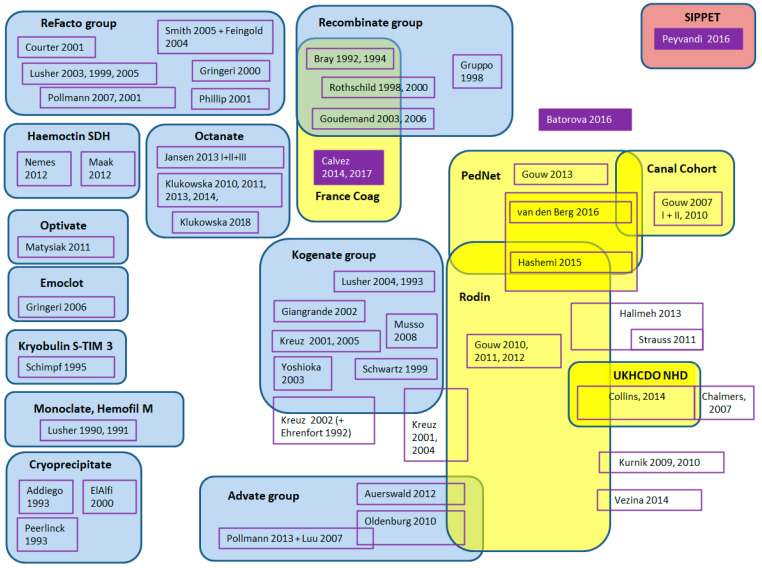
Risk of overlap of individual studies—Studies evaluating a single factor concentrate are summarised under the name of the factor concentrate in blue boxes. Studies originating from registry data are presented in yellow boxes and prospective trials in pink boxes. The three remaining relevant studies following the parameters demanded in the ClinGL are shown in purple.

The potential for overlap of the individual studies was evaluated depending on centres respectively countries where the trial was performed, recruitment period, and investigators. The assumed potential overlap of study populations is shown in Figure 4.

The figure demonstrates that studies evaluating a single factor concentrate often generate several publications over time. If such publications are to be included in a meta-analysis, it must be clear that the patient numbers cannot be added up but only supplemented. Determining whether publications are based on the same cohort of patients can be easier if the same authors publish the supplementary information (see Octanate) and difficult if not impossible to find out if a group of researchers is conducting the study and the first authors frequently change (see ReFacto, Kogenate, Recombinate, Advate). It could also be shown that data from registries are more often used in various publications, either by extracting and publishing data from a single factor product or by performing an analysis of all factor products included in the registry. It is important that in a literature meta-analysis, these numbers are not added up, but that care is taken to detect and avoid possible overlap.

### 3.5. Time of Publication and Study Duration

**Figure 5:**
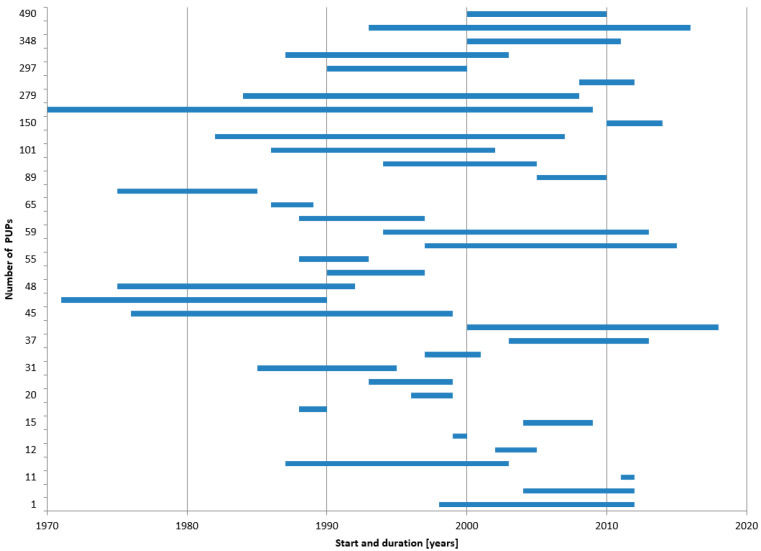
Previously untreated patients (PUP) studies—Number of patients, study start and study duration. Shown are 37 of the 38 publications (for one, the duration could not be found) with the number of recruited patients, the year of study start, and study duration. The publications and underlying data can be found in the Supplement to this paper (File S4).

Recruitment for PUP studies took, on average, more than 10 years from study start to completion (Figure 5).

Such long periods have some drawbacks. First, HA treatment has made major improvements in the period covered here, including changes in the treatment regimen from on-demand to low or high dose prophylactic therapy. Second, laboratory methods varied by time, e.g., the Nijmegen modification of the Bethesda Assay was introduced. Third, several HA-related definitions have been refined, e.g., definitions of low and high titre as well as transient inhibitors or definitions of bleeding episodes. Finally, yet importantly, the concept for inhibitor testing was modified from a weekly or monthly testing schedule to a concept based on exposure days, which has been proven to describe the most vulnerable period for PUP inhibitor development more precisely. Hence, results from PUP studies with such long recruitment periods must be interpreted with caution because of changing methodological and medical standards during the conduct of the study. This is of particular concern if the therapy of later recruited PUPs is either no longer state-of-the-art or must be adapted by study amendments, so that data of recruited patients may differ dependent on their recruitment date.

### 3.6. Analysis Results

**Figure 6:**
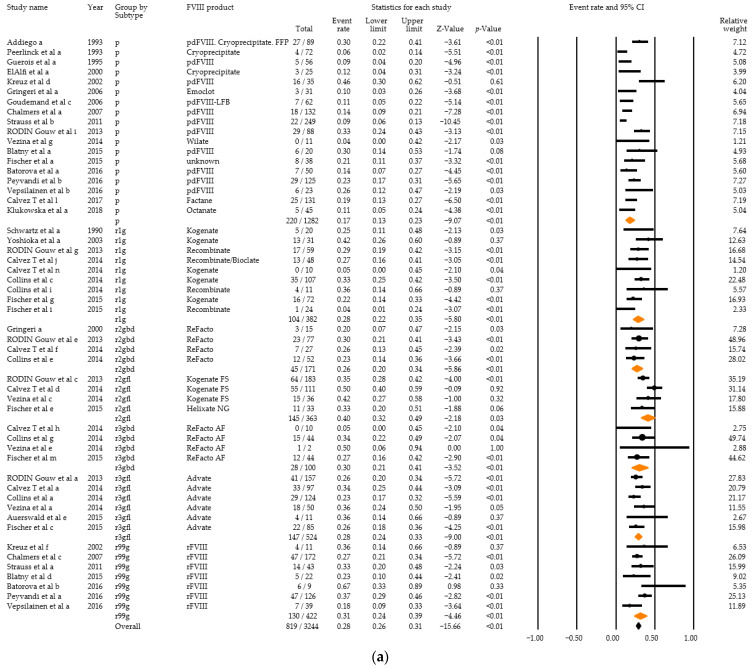

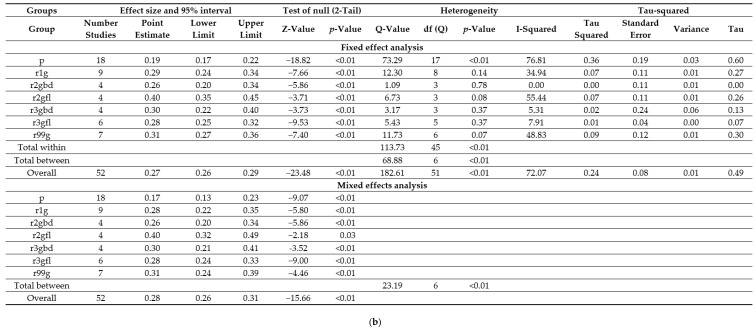
Analysis results for high severity disease and any titre inhibitor patients of the 22 relevant studies: Forest plot (**a**), Basic statistics (**b**). p = plasma-derived; r1g = recombinant 1, generation full length; r2gfl = recombinant 2, generation full-length; r2gbd = recombinant 2, generation B domain deleted; 3gfl = recombinant 3, generation full-length; r3gbd = recombinant 3, generation B domain deleted; rg99 = recombinant not further specified.

Twenty-two studies with high severity disease and any titre inhibitor development in a total of 819 PUPS among 3244 PUPS recruited were eligible for inclusion in the meta-analysis. Combined event rates for any titre inhibitor development ranged from 17% for plasma-derived products to 26–40% for recombinant products (Figure 6a). The combined event rate accounted for 28% (95% CI: 26–31%). Overall heterogeneity was high (Q = 182.61, df = 51, *p* < 0.01, I^2^ = 72.07), and there was a statistically significant difference between the subgroups (Q = 23.19, df = 6, *p* < 0.01) (Figure 6b).

**Figure 7:**
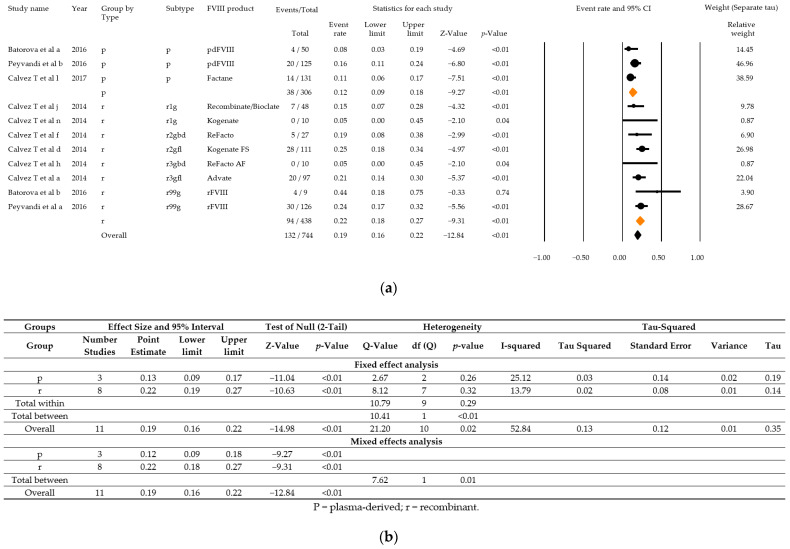
Analysis results for high severity disease and high titre inhibitor patients of the three relevant studies that fulfil all parameters as demanded in the ClinGL: Forest plot (**a**), Basic statistics (**b**). p = plasma-derived; r recombinant; r1g = recombinant 1, generation full length; r2gfl = recombinant 2, generation full-length; r2gbd = recombinant 2, generation B domain deleted; 3gfl = recombinant 3, generation full-length; r3gbd = recombinant 3, generation B domain deleted; rg99 = recombinant not further specified.

Within the scope of the second meta-analysis, three studies with a total of 132 PUPS with high severity disease and high titre inhibitor development among 744 PUPS recruited were included. Combined event rates for high titre inhibitor development were estimated to be 12% (95% CI: 9–18%) for plasma-derived products and 22% (95% CI: 18–27%) for recombinant products (Figure 7a,b). The combined event rate accounted for 19% (95% CI: 16–22%). Overall heterogeneity was high (Q = 21.20, df = 10, *p* = 0.02, I^2^ = 52.82), and there was also a statistically significant difference between the subgroups (Q = 7.62, df = 1, *p* = 0.01). Estimates for different generations of recombinant products were not calculated due to the small number of studies eligible per group.

## 4. Discussion

This systematic review included 38 studies published between 1990 and 2018. The two meta-analyses performed suggested a difference in PUP inhibitor rates between plasma-derived and recombinant FVIII products consistent with other reviews [8,9,10]. Considering the high heterogeneity, this finding has to be interpreted with caution. In the field of haemophilia research and clinical practice, substantial transformation in methods and knowledge has occurred over the last 40 years. Hence, considerable differences in clinical trials (CTs) carried out in the last decades are not surprising. Triggered by blood products contaminated with HIV and hepatitis C and B at the end of the 1980s and beginning of the 1990s, CTs initially focused on the effective heat inactivation of plasma-derived blood products and the associated virus safety and then, with the emergence of recombinant factor concentrates, on the safety of this new product class. The detection of inhibitors became more and more important, and a refinement of the laboratory methods, on the one hand, and the relevant observation period with the greatest risk of inhibitor development, on the other hand, led to more sophisticated CT designs. The results of early CTs in PUPs led to additional knowledge regarding potential confounding factors, such as the family history of inhibitor development, underlying mutation, and intensity of treatment. In addition, the development of Nijmegen’s modification of the Bethesda assay in 1995 [58] led to more precise laboratory results and the possibility to reliably differentiate between high and low titre inhibitors. DNA sequencing and its widespread use after automation of the procedure opened up a completely new field of knowledge that allowed linking the type of mutation with the manifestation and severity of HA as well as the potential risk for inhibitor development. This resulted in numerous CTs and observational studies with different study designs, duration of follow-up or frequency of inhibitor testing, including patients with various characteristics and risk factors, and finally impairing a head-to-head comparison of results.

The ClinGL, which came into effect in 2012, was implemented to achieve greater harmonisation and a higher grade of comparability between CTs. However, interpretation of results derived from small, uncontrolled clinical trials is challenging even in the case of harmonisation of study conduct and methodology due to unmeasured potential confounding related to various risk factors for inhibitor development. For seven out of nine FVIII products that seek approval during or after the ClinGL came into effect, CTs enrolling PUPs were initiated, and some of them are still ongoing, while others were completed or terminated (see supplement File S5).

RCTs allocating participants by chance to one or more treatment groups are usually considered as the gold standard to evaluate the efficacy of a therapy. In rare diseases, such as HA, the conduct of RCTs is, however, hampered by methodological and data constraints, such as the limited number of eligible patients (e.g., PUPs) and the geographic dispersion of patients [55]. These constraints of CTs in PUPs finally led to the revision of the ClinGL, waiving the obligation to perform clinical trials in PUPs for marketing authorisation [16].

The revised ClinGL states that both PUPs and previously treated patients (PTPs) should be encouraged to enroll in disease-specific registries [16]. Because a variety of national and international HA registries exist, the ClinGL defined a core parameter set, allowing for data merging [16]. The core data set includes administrative information as well as information on demographics, anamnesis, treatment, inhibitor formation, and relevant concomitant events. Strictly speaking, a registry is a longitudinal prospective observational study. It is able to overcome the “5 too’s” of RCTs that have already been described by Rogers in 1991: too few subjects, too simple in terms of comorbidities or concomitantly administered drugs, too median aged subject pool, too narrow a definition of the clinical condition, and too brief period for evaluation [59]. Whereas registry data better reflects real life, the registry’s informative value is restricted by crucial pitfalls, such as selection bias and confounding. Selection bias may arise if participants are not allocated by chance to the study groups. In her recent publication [60], Kathelijn Fischer identified two causes of bias, in particular, when comparing inhibitor incidences from different sources: the risk of patient selection (e.g., risk factors and/or treatment strategies) and information bias (e.g., definitions and/or follow-up). For example, the attending physicians might assign a patient with a very severe clinical picture or pre-existing risk factors for the development of an inhibitor to a more “promising” or “safe” therapy scheme. In addition, recruiting a control group might be a challenge in observational studies since cases and controls should be taken from the same population. Furthermore, selection bias may also be caused by the selective non-participation of individuals. In this case, the reasons for non-participation should be recorded and discussed. Bias due to confounding occurs when a spurious association arises due to a failure to fully adjust for factors related to both the risk factor and outcome. A confounding variable is associated with the exposure and disease under study and may influence both the supposed cause and the supposed effect. Researchers can prevent or correct for confounding by restricting the analysis to individuals without a known confounder by matching, stratification, and using specific statistical procedures (e.g., multiple logistic/cox regression analysis) [61]. However, there is always a risk that so-called “unmeasured” confounding (unknown and/or not recorded confounders) may affect the results of an observational study.

Overall, the risk of bias for the 38 primary publications was rated low to serious. However, for some studies, little or no information was available regarding bias (Table 1). Due to the lack of respective information, it was not possible to assess the selection bias that may result from the requirement to enroll only PUPs in CTs. In fact, this often leads to the exclusion of patients born in families with a negative family history of HA as well as patients who needed intensive treatment at first exposure. The possible impact of the following known confounders could not be excluded: Ethnicity was described in 28/38 studies, underlying mutation (“genetics”) in 19 studies, family history of HA in 21 and family history of inhibitor development in 28 studies. Cox proportional hazard models considering potential confounders were applied only in 11 of 38 primary publications.

Of course, there are established standards and guidelines for the conduct and reporting of observational studies in general, addressing research plan, methods, statistical analysis, discussion, and conclusion that allow addressing and controlling for the most important limitations of these studies [61], but these documents are only partly helpful. Apart from HA-specific registries sharing the same core data set, a common set of agreed minimum standards and guidelines for conducting observational studies on FVIII inhibitor development is required to increase the likelihood of high-quality research and robustness of observational study results. Even with the advent and establishment of novel treatments for HA, such as bypassing agents, FVIII-mimicking agents and gene therapy, these standards and guidelines may help to harmonise research on therapies for HA. Heterogeneity of studies in rare diseases, such as HA, is a major problem in general. As another example, a comparison of studies examining the cost-utility of prophylaxis versus on-demand therapy in HA found that these studies yielded remarkably different results [62]. As noted by the authors, cooperation among key stakeholders is essential to resolve issues outstanding from evidence-based and experimental data, which applies likewise to efficacy and safety assessment of current and emergent HA therapies.

## 5. Conclusions

To conclude, in rare diseases, the number of patients that can be recruited in CTs is limited, and thus, randomised clinical trials for individual products are challenging, in particular, for investigating potential individual risks of individual products. With a 1:1 randomisation and increasing the inhibitor rate from 30% in control subjects to 40% under investigational drug, 356 participants would have to be recruited per group to reject the null hypothesis with 80% power and a Type I error probability of 5%. Therefore, enrolling a sufficient number of patients required for a robust statistical analysis can take a long time. Meanwhile, both laboratory standards and detailed knowledge of the disease may evolve, which ultimately may produce findings that, at the end of the study, are no longer considered state-of-art. Furthermore, additional parameters not collected within the scope of the study may become relevant. Performing a meta-analysis of heterogenic studies to overcome these issues is like shaving cats, dogs, alpaca, rabbits, and sheep and wondering in the end why there is not enough mohair for a pullover.

Finally, what longer-term perspective emerges from this work? This exercise led to a result that is comparable to the results of numerous previous studies and analyses. However, considering the numerous limitations addressed above, these findings are not supportive of decision-making. Which path must be taken to obtain results that are necessary for regulatory decisions? A large network of well-managed registries is seen as the only realistic option, as this would allow the monitoring and investigation of at least those factor concentrates that are actually used in the respective patient groups. Of course, these prerequisites apply equally to other new therapeutic options that are striving to reach the market, such as monoclonal antibodies or gene therapy. Therefore, a commitment of regulators is necessary to carry out a regular analysis or review of the data derived from registries. To achieve this, a well-planned collaboration of registry operators and regulators involving patients and scientists is required, as well as a prior determination and definition of the regulatory measures to be taken when predetermined triggers are exceeded.

## Figures and Tables

**Table 1:** Risk of bias in individual studies.

Publication	Risk of Confounding Bias	Risk of Selection Bias	Risk of Bias Due to Departure from Interventions	Risk of Bias Due to Missing Data	Risk of Information Bias	Risk of Reporting Bias
Risk of bias for interventional studies						
Auerswald et al., 2012 [23]	low	moderate	moderate	low	moderate	low
Auerswald et al., 2015 [24]	moderate	low	low	low	moderate	low
Batorova et al., 2016 [21]	moderate	low	low	moderate	moderate	low
Biasi et al., 1994 [25]	serious	low	no information	moderate	moderate	low
Bray et al., 1994 [26]	serious	low	low	moderate	moderate	low
Courter et al., 2001 [27]	moderate	low	low	moderate	moderate	low
ElAlfi et al., 2000 [28]	serious	low	low	moderate	serious	low
Gouw et al., 2013 [5]	low	low	low	low	moderate	low
Guérois et al., 1995 [29]	moderate	no information	no information	no information	moderate	low
Klukowska et al., 2018 [30]	low	low	low	low	low	low
Kreuz et al., 2005 [31]	low	low	low	low	low	low
Kreuz et al., 2002 [32]	moderate	moderate	low	moderate	moderate	low
Lusher and Salzmann 1990 [33]	moderate	low	low	serious	serious	low
Lusher et al., 2004 [34]	moderate	low	moderate	moderate	moderate	moderate
Matysiak et al., 2011 [35]	moderate	low	low	moderate	serious	low
Peyvandi et al., 2016 [7]	low	low	moderate	low	low	low
Schwartz et al., 1990 [36]	serious	low	no information	low	serious	moderate
Yee et al., 1997 [37]	serious	low	no information	moderate	serious	moderate
Yashioka et al., 2003 [38]	serious	no information	low	low	moderate	low
Risk of bias for observational studies						
Addiego et al., 1993 [39]	serious	no information	no information	no information	no information	low
Blatny et al., 2003 [40]	serious	moderate	moderate	moderate	moderate	low
Calvez et al., 2014 and 2018 [22,41]	low	low	low	low	moderate	low
Chalmers et al., 2007 [42]	moderate	low	no information	no information	no information	low
Collins et al., 2014 [43]	low	low	low	low	low	low
Fischer et al., 2015 [44]	serious	no information	no information	low	no information	low
Goudemand et al., 2006 [45]	moderate	moderate	no information	no information	no information	low
Gouw et al., 2007 [46]	low	low	low	low	no information	low
Gringeri et al., 2000 [47]	serious	no information	no information	no information	no information	no information
Gringeri et al., 2006 [48]	serious	low	no information	moderate	moderate	low
Kurnik et al., 2009 [49]	serious	no information	no information	no information	no information	low
Maak et al., 2012 [50]	serious	no information	no information	no information	no information	low
Mancuso et al., 2012 [51]	low	low	low	moderate	serious	low
Musso et al., 2008 [52]	moderate	low	moderate	moderate	moderate	low
Oldenburg et al., 2010 [53]	moderate	no information	no information	no information	no information	low
Peerlinck et al., 1993 [54]	serious	low	no information	no information	no information	low
Strauss et al., 2011 [55]	moderate	no information	low	no information	moderate	low
Vepsäläinen et al., 2016 [56]	low	no information	moderate	moderate	no information	low
Vézina et al., 2014 [57]	moderate	low	low	low	moderate	low

## Data Availability

The data presented in this study are available in the article (Figure 6 and Figure 7) and in the supplement (Files S2 and S4).

## Supplementary Materials

The following are available online at https://www.mdpi.com/1660-4601/18/1/225/s1, File S1: Example of a full search in Medline; File S2: An overview of all 80 publications and the assessment process that resulted in the generation of Figure 3; File S3: An overview of the rating process that is summarised in Table 1; File S4: The publications and underlying data that is shown in Figure 5; File S5: Overview of FVIII products that seek approval during or after the ClinGL came into effect.
