# Supplementary material for: Epidemiological Challenges in Rare Bleeding Disorders: FVIII Inhibitor Incidence in Haemophilia A Patients—A Known Issue of Unknown Origin
Source: Int J Environ Res Public Health. 2020 Dec 30;18(1):225. doi: 10.3390/ijerph18010225 (PMC7795862; doi:10.3390/ijerph18010225)
Supplement: Supplementary file 1 [file ijerph-18-00225-s001.zip › Supplement 1.docx]

**Example of a full search in Medline**

#1. ("previously untreated"[All Fields] OR "minimally treated"[All Fields]) AND ("hemophilia a"[MeSH Terms] OR "haemophilia a"[All Fields])

#2. "factor viii"[MeSH Terms] AND ("antibody"[All Fields] OR "antibodies"[MeSH Terms])

#3. "plasma"[MeSH Terms] AND "derived"[All Fields] AND "factor viii"[MeSH Terms]

#4. "recombinant"[All Fields] AND "factor viii"[MeSH Terms]

#5. "advate"[All Fields] OR "f8 protein human"[Supplementary Concept] OR "f8 protein human"[All Fields] OR "kogenate"[All Fields] OR "recombinant factor VIII SQ"[Supplementary Concept] OR "recombinant factor VIII SQ"[All Fields] OR "ReFacto"[All Fields] OR "Helixate"[All Fields] OR "Humate-P"[All Fields] OR "Profilate"[All Fields] OR "Alphanate"[All Fields] OR "Koate"[All Fields] OR "Hemophil"[All Fields] OR "Hemofil"[All Fields] OR "Monoclate"[All Fields] OR "Bioclate"[All Fields] OR "Recombinate"[All Fields] OR "ReFacto"[All Fields]

#6. #1 or#2

#7. #3 OR #4 OR #5

#8. #6 OR #7
