# Supplementary material for: Epidemiological Challenges in Rare Bleeding Disorders: FVIII Inhibitor Incidence in Haemophilia A Patients—A Known Issue of Unknown Origin
Source: Int J Environ Res Public Health. 2020 Dec 30;18(1):225. doi: 10.3390/ijerph18010225 (PMC7795862; doi:10.3390/ijerph18010225)
Supplement: Supplementary file 1 [file ijerph-18-00225-s001.zip › Supplement 2.docx]

| **Publication** | **Factor VIII Product** | **Study design Study  period Follow up (FU)** |  | **Definition FVIII level haemophilia A** | **Number recruited PUP/MTP + Severity** | **Inhibitor test Bethesda assay, + Nijmegen modification, other** | **Frequency of testing** | **Parameters according to ClinGL fulfilled?** | | | | |
| --- | --- | --- | --- | --- | --- | --- | --- | --- | --- | --- | --- | --- |
|  |  |  | **Study location (number of sites)** |  |  |  |  | **>= 50 PUPs (no MTPs)** | **>= 50 ED FU** | **Severity <1%** | **Bethesda (or later than ca. 1995: Nijmegen Modification)** | **Inhibitor testing frequency (** |
| **(Addiego et al. 1993)** | pdFVIII Cryo-precipitate FFP | retrospective, uncontrolled, 1975-1985  FU: 5 years or 30 exposure days | USA (7) | severe: < 1 % | 89 PUP | Bethesda assay | at least annually | y | n | y | y | n |
| **(Auerswald et al. 2012)** | Advate | prospective, uncontrolled 2004-2009 FU: 3 years or 75 EDs | USA (13), France (4) Germany (2), Austria (1), Canada (1), Italy (1), Spain (1), UK (1), | severe: < 1%,  moderately severe:1–2% | 18 PUP/ 37 MTP, 53 severe, 1 moderate, 1 mild | Bethesda test Nijmegen assay | ≤10, 20,or 30 EDs | n | y | y | y | y |
| **(Auerswald et al. 2015)** | Advate | prospective, historically controlled 2011-2012 FU: 50 EDs | Germany (4), Russia (3), USA (2), Poland (2), Austria (1), Bulgaria (1), Canada (1), Czech Republic (1), Lithuania (1), Netherlands (1), Serbia (1), Spain (1) | severe: < 1 % | 11 PUP/ 8 MTP | Nijmegen modified Bethesda assay | at infusion number 3, 6, 10, 15, 20, 30, 40 and 50 | n | y | y | y | y |
| **(Batorova et al. 2016)** | pdFVIII rFVIII | prospective, uncontrolled 1997-2015 FU: up to 50 and 150 EDs | Slovakia | severe: < 1 % | 59 PUP: pdFVIII: 50  rFVIII: 9 | Both standard Bethesda method and Nijmegen modification | every 4 to 5 exposure days (EDs) during the first 20 EDs, then every 10 and 20 EDs up to 50 and 150 EDs | y | y | y | y | y |
| **(Biasi et al. 1994)** | pdFVIII | prospective, uncontrolled 1975-1992 FU: no FU in ED defined | Italy | severe and moderate: < 1 % to =< 5% | 64 PUPs, 48 severe, 16 moderate | Bethesda assay | repeatedly when clinically indicated or at least annually | y | n | y | y | n |
| **(Blatny et al. 2015)** | pdFVIII rFVIII | retrospective, uncontrolled, 2003-2013 FU: at least 100 EDs | Czech Republic | no definition provided | 86 PUP rFVIII: 45 (22 severe, 11 moderate, 12 mild) pdFVIII: 41(20 severe, 5 moderate, 16 mild) | Nijmegen modified Bethesda assay | every 5 exposure days (EDs) during the first 20 EDs, then every 10 EDs up to 50 Eds and then 6 monthly | y | y | unknown | y | y |
| **(Bray et al. 1994** **(Bray 1992; Gruppo R. et al. 1998; Rothschild et al. 2000; Goodeve et al. 2000)** | Recombinate | prospective, uncontrolled 1990-1997 FU: minimum of 24 months or 50 rAHF ED | USA (26), Denmark (2), France (2), Italy (1), Germany (1) | severe: < 1 %,  moderate: ≤ 2% | 69 PUP, 2 MTP, 55 severe , 14 moderate | Bethesda | every 3 month | y | y | y | y | n |
| **(Calvez et al. 2014; Calvez et al. 2018)** | Recombinate/Bioclate, Kogenate/Helixate, Refacto, Kogenate FS/Helixate NexGen, Advate, ReFacto AF/Xyntha, Factane | retrospective, uncontrolled 1993-2014 ( -2016) FU: the first 75 EDs | France | severe: < 1 % | 490 PUP | Bethesda method or the Nijmegen modified assay | results show: On average, these assays were performed every 6.3 EDs during the first 25 EDs and every 9.9 EDs during the overall follow-up period | y | y | y | y | y |
| **(Chalmers et al. 2007)** | pdFVIII rFVIII | retrospective, uncontrolled 1987-2003 FU: >50 EDs | UK | severe: < 1 % | 348 PUP | Bethesda assay | Monitoring for inhibitory antibodies was performed on a regular basis at least every 3–6 months | y | y | y | y | n |
| **(Collins et al. 2014)** | Advate Kogenate Bayer/Helixate ReFacto Refacto AF Recombinate | retrospective, uncontrolled 2000-2011 FU: 75 EDs | UK | severe: <1% | 407PUP  Advate:172  Kogenate Bayer/Helixate: 128 ReFacto: 52  Refacto AF: 44  Recombinate: 11 | "measured locally using standard Bethesda or Nijmegen assays" | not reported | y | y | y | y | unknown |
| **(Courter and Bedrosian 2001)(Lusher et al. 2003)**  **(Lusher and Roth 2005; Philipp CS et al. 2001; Pollmann et al. 2007; Pollmann H 2001; Smith et al. 2005; Feingold JM et al. 2004; Lusher JM et al. 1999)** | Refacto | prospective, uncontrolled 1994-2005 FU: 50ED or up to 5 years | Germany  (3), Spain (6), Austria (3), Holland (2), UK (7), France (7), Italy (3), Sweden (1),  Denmark (2), Switzerland (1), Hungary (1), South  Africa (1), Turkey (1), Belgium (1), USA (10) | severe: <2% | 101 PUP | Bethesda assay | not specified | y | y | n | y | unknown |
| **(ElAlfy et al. 2000)** | Cryoprecipitate | prospective, uncontrolled 1996 -1999  FU: 3 years | Egypt | severe: < 1 % | 25 PUP | Bethesda | twice a year | n | unknown | y | y | n |
| **(Fischer et al. 2015)** | Advate,   Helixate NexGen,  Kogenate Bayer, Recombinate, Refacto,Refacto AF, PD products | retrospective, uncontrolled  (EUHASS) 2008 - 2012 FU: >50EDs | EU (26) | no definition provided | 297 PUP all severe | No information | not specified | y | y | unknown | unknown | unknown |
| **(Goudemand et al. 2006; Rothschild et al. 1998; Goudemand J et al. 2003; Bray et al. 1994)** | Recombinate pdFVIII-LFB Kogenate | retrospective,  uncontrolled 1986-2002  FU: median observation time 108 ED | France (23) | severe: <1 % | 148 PUP all severe | Bethesda | not specified | y | n | y | y | unknown |
| **(Gouw et al. 2007a)**  **(; Gouw et al. 2007b; Gouw SC et al. 2010; Mauser-Bunschoten et al. 2007; Mauser-Bunschoten et al. 2001; van der Bom et al. 2003)** | PdFVIII: 135, RdFVIII: 181, | retrospective, uncontrolled  (CANAL) 1990-2000 FU: up to 50 ED or until inhibitor development | EU (13), Canada (1) | severe: <2% | 316 | Bethesda | not specified | y | y | n | y | unknown |
| **(Gouw et al. 2013a)**  **(Gouw et al. 2013b; Gouw S et al. 2010; Gouw S et al. 2012; Gouw SC et al. 2011; van den Berg et al. 2016)** | 1 pdFVIII 2 Recombinate 3 Kogenate FS 4 ReFacto 5 Advate | prospective, uncontrolled  (RODIN)  2000-2010 FU: 75 ED | 29 centers in Europe, Israel and Canada | severe: <1 % | 574 PUP | Bethesda | not specified | y | y | y | n | unknown |
| **(Gringeri et al. 2006)** | Emoclot | retrospective,  uncontrolled 1987–2003 FU: at least 20 EDs | Italy (13) | severe: <1% moderate: 1–5% | 31 PUP (12 severe, 17 moderate)68 MTP (57 severe, 11 moderate) | Bethesda | every 5–10 EDs | n | n | y | y | y |
| **(Gringeri A et al. 2000)** | BDDrFVIII Refacto | Retrospective, uncontrolled 1999-2000 FU: median 20 EDs | Italy (32) | severe: <2%,  non-severe: ≥2% | 17 PUP: 15 PUP severe, 2 PUP non-severe | Bethesda | not specified | n | n | n | y | unknown |
| **(Guérois et al. 1995)** | highly purified pdFVIII | prospective, uncontrolled 1988-1993 FU: median 26 | France (13) | severe: <1% | 56 PUP | 1991-1993: Bethesda, <1991 = APTT (n=10) | every 3 to 6 month but at least once a year | y | n | y | n | n |
| **(Kurnik K et al. 2009)**  **(Halimeh et al. 2013; Kurnik et al. 2010)** | pdFVIII rFVIII | Prospective, uncontrolled, 1982-2007  FU: 200 ED | Germany (5) | severe/no definition provided | 150 PUPs | Bethesda assay or Nijmegen modification | at least monthly to 3 monthly | y | y | n | y | n |
| **(Klukowska et al. 2018)**  **(Klukowska A et al. 2014; Klukowska A et al. 2010; Klukowska A et al. 2011; Klukowska A et al. 2013; Klukowska et al. 2011; Jansen M et al. 2013a, 2013b; Jansen M et al. 2013c)** | Octanate | prospective, uncontrolled 2000-2018 FU: 100 EDs | Poland, Czech Republic, Russia | severe: < 1 %  moderate < 2 % | 51 PUP recruited 45 PUP: severe 4 PUP: moderate (excluded) 2 PUP :<20 Eds (excluded) | Bethesda assay with Nijmegen modification | every 3-4 exposure days (ED 1-20), every 10 EDs (ED 21-100),  . | y | y | n | y | y |
| **(Kreuz et al. 2005)**  **(Kreuz et al. 2001; Giangrande 2002)** | Kogenate FS | Prospective, uncontrolled,  1997-2001  FU: median 114 EDs | US (13), Germany (4), Spain (3), France (3), UK (3), Sweden (2), Denmark (2), Portugal (1), Israel (1) | severe: < 2 % | 37 PUP, 24 MTP: 49 severe, 12 moderate | Bethesda assay with Nijmegen modification | every 3rd-4th ED until the 20th ED, then after every 10th ED until the 50th ED | n | n | n | y | y |
| **(Kreuz et al. 2002)****( Ehrenforth S et al. 1992)** | pdFVIII rFVIII | prospective, uncontrolled 1976-1999 FU: pdFVIII: median 290 ED; rFVIII: median 59 | Germany | severe: <1% moderate: 1 to 5% | 72 PUP Pd FVIII: 51, rFVIII: 21,  Severe: 46, moderate: 26 | Bethesda method | every 3rd to 5th ED during the first 20 ED, every 10th ED until the 200th ED | y | n | y | y | y |
| **(Lusher and Salzman 1990; Lusher 1991)** | Monoclate Hemofil | prospective, uncontrolled, 1986-1989, FU: surveillance at 6-month intervals | US (10), UK (2), Netherlands (1), Israel (1) | no definition provided | Monoclate 38 (19 PUP, 19 MTP), Hemofil: 51 (48 PUP, 3 MTP), Severity not clear | Bethesda | at baseline , then every six month | y | unknown | unknown | y | unknown |
| **(Lusher et al. 2004; Lusher et al. 1993)** | Kogenate | prospective, uncontrolled 1988 –1997 FU: up to 75 ED | USA (15), Canada (1), Germany (4), Italy (3), Spain (3), Sweden (2) | severe: <2 | 100 PUP, 2 MTP,  65 severe, 16 moderate, 21 mild | Bethesda (without Nijmegen) | before study entry, then every three month | y | y | n | y | n |
| **(Maak B. et al. 2012)** | Haemoctin SDH | prospective, uncontrolled 1998-ongoing FU: mean observation period of 53 months | Germany (12) | Severe: <2 | 6 PUP | no information | at baseline, then at least once a year | n | unknown | n | unknown | n |
| **(Mancuso et al. 2012)** | pdFVIII  rFVIII | retrospective, uncontrolled 1922 - 2009 FU: 150 EDs or detection of inhibitors | Italy (3) | severe: <1% moderate: 1%–4% | 377 analysed: 279 PUP and 98 MTP, 318 severe | Bethesda assay ; Nijmegen modification | frequency not specified | y | y | y | y | unknown |
| **(Matysiak et al. 2011)** | Optivate | prospective, uncontrolled period not clear FU: 26 weeks of treatment | Poland (5) | severe: <1% | 1 PUP | other + Bethesda/Nijmegen assay | every three month | n | unknown | y | y | n |
| **(Musso et al. 2008)** | Kogenate | prospective, uncontrolled 2002-2005 FU: up to 24 months, mean (± SD) of 187 (121)  EDs | Austria (2), Belgium (2), Denmark (1), France (24), Greece (2), Italy (6), The Netherlands (6), Spain (4), Sweden (3), Switzerland (2) | Severe: <2% | 13 PUPs | Bethesda | frequency not specified | n | n | n | n | unknown |
| **(Oldenburg et al. 2010; Luu et al. 2007; Pollmann et al. 2013)** | Advate | prospective, uncontrolled 2004-2012 FU: 12 months following study entry | US, Austria, Belgium, Denmark, France, Gemrany, Greece, The Netherlands, Spain, Sweden, Switzerland, UK | severe: <1% moderately severe: 1% -≤2%  moderate: 2% - ≤5%   mild:>5% | 11 severe to moderate PUP  1 mild PUP (Those with 0–3 ED were considered PUPs) | Bethesda | frequency not specified | n | unknown | y | n | unknown |
| **(Peerlinck et al. 1993)** | Cryoprecipitate | retrospective, uncontrolled 1971-1990  FU: <100 ED | NL (1) | severe: <1% | 72 PUP (67 treated) severe: 48  moderate: 10  mild: 14 | other and Bethesda assay | frequency not specified | y | y | y | y | unknown |
| **(Peyvandi et al. 2016)** | rFVIII (Advate, Kogenate FS, Recombinate, ReFacto AF); pdFVIII (Alphanate, Emoclot, Factane, Fanhdi) | prospective, controlled 2010- 2014 FU: 50 consecutive EDs or 3 years | Italy, India, Iran, US, Mexico, Brazil, Chile, Argentina, South Africa, Spain, Saudi-Arabia, Austria, Turkey, Netherlands | severe: <1% | 251 PUP: pdFVIII: 125 rFVIII: 126 | Bethesda assay with the Nijmegen modification local and central laboratory | every 3 to 4 exposure days during the first 20 infusions, then every 10 exposure days or every 3 months | y | y | y | y | y |
| **(Schwartz et al. 1990)** | Kogenate | prospective, uncontrolled 1988 – 1990 FU: not specified | US, EU, Japan | severe: <1% moderate: 1%-5% mild: > 5% | 20 PUP (severe), 1 MTP (moderate) | Bethesda | approx. every 12 weeks | n | unknown | y | y | n |
| **(Strauss et al. 2011)**  **(Halimeh et al. 2013)** | Refacto/Refacto AF; Kogenate FS/Helixate NG, full length second generation rFVIII or B domain deleted products, pdFVIII concentrates | retrospective 1984-1995 prospective 1996-2008, uncontrolled FU: at least 75 EDs | Israel | severe: <1% | 292 PUP:  pdFVIII: 249 rFVIII: 43 | Bethesda method | 1984-1996: once a year,>1996: at least every 6 month | y | y | y | y | n |
| **(Vepsäläinen et al. 2016)** | Recombinate, Kogenate/Helixate, Refacto, Advate, ReFacto AF, Amofil, Haemate, Cryoprecipitate | retrospective, uncontrolled 1994 - 2013 FU: at least 75 EDs | Finland (5) | severe: <1% | 62 PUP:  rFVIII: 39 pdFVIII: 23 | Bethesda assay | every 6 month or upon clinical suspicion | y | y | y | n | n |
| **(Vézina et al. 2014)** | Advate, Kogenate FS or Helixate, Wilate, RefactoAF/ Xyntha | retrospective, uncontrolled 2005-2010 FU: at minimum 20 ED | Canada (26) | severe: <1% | 99 PUP | Bethesda assay or Nijmegen modification | frequency not specified | y | n | y | y | unknown |
| **(Yee et al. 1997)** | pdFVIII | prospective, uncontrolled 1985-1995 FU: median of 200 ED | UK (2) | severe: <2% | 37 PUP | Bethesda assay or other | every three month, every four months, biannually or annually | n | n | n | y | n |
| **(Yoshioka et al. 2006)** | Kogenate | prospective, uncontrolled 1993-1999 FU: 11 to 80 months | Japan (33) | severe: <1% moderate: 1%-5% mild: > 5% | 43 PUP: 31 severe 9 moderate 3 mild | Bethesda assay | at inclusion and 3, 6, 9, 12, 18 and 24 months after inclusion | n | n | y | y | n |
|  |  |  |  |  |  |  |  |  |  |  |  |  |
|  |  |  |  |  |  |  |  | 28 | 23 | 26 | 34 | 11 |
|  |  |  |  |  |  |  |  | 21 | |  | |  |
|  |  |  |  |  |  |  |  | 12 | | |  |  |
|  |  |  |  |  |  |  |  | 9 | | | |  |
|  |  |  |  |  |  |  |  | 3 | | | | |

Publication bibliography

Addiego, J.; Kasper, C.; Abildgaard, C.; Hilgartner, M.; Lusher, J.; Glader, B.; Aledort, L. (1993): Frequency of inhibitor development in haemophiliacs treated with low-purity factor VIII. In *Lancet (London, England)* 342 (8869), pp. 462–464.

Auerswald, G.; Kurnik, K.; Aledort, L. M.; Chehadeh, H.; Loew-Baselli, A.; Steinitz, K.; Reininger, A. J. (2015): The EPIC study. A lesson to learn. In *Haemophilia : the official journal of the World Federation of Hemophilia* 21 (5), pp. 622–628. DOI: 10.1111/hae-12666.

Auerswald, Guenter; Thompson, Alexis A.; Recht, Michael; Brown, Deborah; Liesner, Raina; Guzmán-Becerra, Norma et al. (2012): Experience of Advate rAHF-PFM in previously untreated patients and minimally treated patients with haemophilia A. In *Thrombosis and haemostasis* 107 (6), pp. 1072–1082. DOI: 10.1160/TH11-09-0642.

Batorova, Angelika; Jankovicova, Denisa; Morongova, Anna; Bubanska, Eva; Prigancova, Tatiana; Horakova, Julia et al. (2016): Inhibitors in Severe Hemophilia A. 25-Year Experience in Slovakia. In *Seminars in thrombosis and hemostasis* 42 (5), pp. 550–562. DOI: 10.1055/s-0036-1581107.

Biasi, R. de; Rocino, A.; Papa, M. L.; Salerno, E.; Mastrullo, L.; Blasi, D. de (1994): Incidence of factor VIII inhibitor development in hemophilia A patients treated with less pure plasma derived concentrates. In *Thrombosis and haemostasis* 71 (5), pp. 544–547.

Blatny, Jan; Komrska, Vladimir; Blazek, Bohumir; Penka, Miroslav; Ovesna, Petra (2015): Inhibitors incidence rate in Czech previously untreated patients with haemophilia A has not increased since introduction of recombinant factor VIII treatment in 2003. In *Blood coagulation & fibrinolysis : an international journal in haemostasis and thrombosis* 26 (6), pp. 673–678. DOI: 10.1097/MBC.0000000000000298.

Bray, G. L. (1992): Current status of clinical studies of recombinant factor VIII (recombinate) in patients with hemophilia A. Recombinate Study Group. In *Transfusion medicine reviews* 6 (4), pp. 252–255.

Bray, G. L.; Gomperts, E. D.; Courter, S.; Gruppo, R.; Gordon, E. M.; Manco-Johnson, M. et al. (1994): A multicenter study of recombinant factor VIII (recombinate). Safety, efficacy, and inhibitor risk in previously untreated patients with hemophilia A. The Recombinate Study Group. In *Blood* 83 (9), pp. 2428–2435.

Calvez, Thierry; Chambost, Hervé; Claeyssens-Donadel, Ségolène; d'Oiron, Roseline; Goulet, Véronique; Guillet, Benoît et al. (2014): Recombinant factor VIII products and inhibitor development in previously untreated boys with severe hemophilia A. In *Blood* 124 (23), pp. 3398–3408. DOI: 10.1182/blood-2014-07-586347.

Calvez, Thierry; Chambost, Hervé; d'Oiron, Roseline; Dalibard, Vincent; Demiguel, Virginie; Doncarli, Alexandra et al. (2018): Analyses of the FranceCoag cohort support differences in immunogenicity among one plasma-derived and two recombinant factor VIII brands in boys with severe hemophilia A. In *Haematologica* 103 (1), pp. 179–189. DOI: 10.3324/haematol.2017.174706.

Chalmers, E. A.; Brown, S. A.; Keeling, D.; Liesner, R.; Richards, M.; Stirling, D. et al. (2007): Early factor VIII exposure and subsequent inhibitor development in children with severe haemophilia A. In *Haemophilia : the official journal of the World Federation of Hemophilia* 13 (2), pp. 149–155. DOI: 10.1111/j.1365-2516.2006.01418.x.

Collins, Peter W.; Palmer, Benedict P.; Chalmers, Elizabeth A.; Hart, Daniel P.; Liesner, Ri; Rangarajan, Savita et al. (2014): Factor VIII brand and the incidence of factor VIII inhibitors in previously untreated UK children with severe hemophilia A, 2000-2011. In *Blood* 124 (23), pp. 3389–3397. DOI: 10.1182/blood-2014-07-580498.

Courter, S. G.; Bedrosian, C. L. (2001): Clinical evaluation of B-domain deleted recombinant factor VIII in previously untreated patients. In *Seminars in hematology* 38 (2 Suppl 4), pp. 52–59.

Ehrenforth S; Kreuz W; Scharrer I; Linde R; Funk M; Güngör T et al. (1992): Incidence of development of factor VIII and factir IX inhibitors in haemophiliacs. In *Lancet (London, England)* (339), pp. 594–598.

ElAlfy, M. S.; El Alfy, M. S.; Tantawy, A. A.; Ahmed, M. H.; Abdin, I. A. (2000): Frequency of inhibitor development in severe haemophilia A children treated with cryoprecipitate and low-dose immune tolerance induction. In *Haemophilia : the official journal of the World Federation of Hemophilia* 6 (6), pp. 635–638.

Feingold JM; Smith MP; Littlewood R; Giangrande P; ReFacto STL study group (Eds.) (2004): A post-marketing surveillance study of the safety and efficacy of ReFacto (Moroctocog Alfa, B-domain deleted recombinant Factor VIII) (St. Louis-derived active substance in the treatment and prevension of bleeding episodes in hemophilia A patients. Abstract 3101.5.

Fischer, Kathelijn; Lassila, Riita; Peyvandi, Flora; Calizzani, Gabriele; Gatt, Alex; Lambert, Thierry et al. (2015): Inhibitor development in haemophilia according to concentrate. Four-year results from the European HAemophilia Safety Surveillance (EUHASS) project. In *Thrombosis and haemostasis* 113 (5), pp. 968–975. DOI: 10.1160/TH14-10-0826.

Giangrande, P. L. F. (2002): Safety and efficacy of KOGENATE Bayer in previously untreated patients (PUPs) and minimally treated patients (MTPs). In *Haemophilia : the official journal of the World Federation of Hemophilia* 8 Suppl 2, pp. 19–22.

Goodeve, A. C.; Williams, I.; Bray, G. L.; Peake, I. R. (2000): Relationship between factor VIII mutation type and inhibitor development in a cohort of previously untreated patients treated with recombinant factor VIII (Recombinate). Recombinate PUP Study Group. In *Thrombosis and haemostasis* 83 (6), pp. 844–848.

Goudemand, Jenny; Rothschild, Chantal; Demiguel, Virginie; Vinciguerrat, Christine; Lambert, Thierry; Chambost, Hervé et al. (2006): Influence of the type of factor VIII concentrate on the incidence of factor VIII inhibitors in previously untreated patients with severe hemophilia A. In *Blood* 107 (1), pp. 46–51. DOI: 10.1182/blood-2005-04-1371.

Goudemand J; Rothchild C; Demiguel V; Lambert T; Calvez T (Eds.) (2003): The type of products (plasmatic vs recombinant) may exert an influence on FVIII inhibitor incidence in previously untreated severe Hemophilia A patients (PUPs). Abstract No. 2954.

Gouw, Samantha C.; van den Berg, H. Marijke; Fischer, Kathelijn; Auerswald, Günter; Carcao, Manuel; Chalmers, Elizabeth et al. (2013a): Intensity of factor VIII treatment and inhibitor development in children with severe hemophilia A. The RODIN study. In *Blood* 121 (20), pp. 4046–4055. DOI: 10.1182/blood-2012-09-457036.

Gouw, Samantha C.; van der Bom, Johanna G.; Ljung, Rolf; Escuriola, Carmen; Cid, Ana R.; Claeyssens-Donadel, Ségolène et al. (2013b): Factor VIII products and inhibitor development in severe hemophilia A. In *The New England journal of medicine* 368 (3), pp. 231–239. DOI: 10.1056/NEJMoa1208024.

Gouw, SC.; van der Bom, JG.; Auerswald, G.; Escuriola Ettinghausen, C.; Tedgård, U.; van den Berg, H. M. (2007a): Recombinant versus plasma-derived factor VIII products and the development of inhibitors in previously untreated patients with severe hemophilia A. The CANAL cohort study. In *Blood* 109 (11), pp. 4693–4697. DOI: 10.1182/blood-2006-11-056317.

Gouw, SC.; van der Bom, JG.; van den Berg, H. M. (2007b): Treatment-related risk factors of inhibitor development in previously untreated patients with hemophilia A. The CANAL cohort study. In *Blood* 109 (11), pp. 4648–4654. DOI: 10.1182/blood-2006-11-056291.

Gouw S; Mancuso E; Santagostino E; van den Berg HM; van der Bom J (Eds.) (2010): Determinants of high titer inhibitor development: The Canal study. Poster 17P33 (Haemophilia, 16 (Suppl. 4)).

Gouw S; van den Berg H; van den Bom J (Eds.) (2012): Factor VIII prophylaxis and inhibitor development in previously untreated patients with severe hemophilia A: The RODIN study. Poster PO-WE-103 (Haemophilia, 18 (Suppl. 3)).

Gouw SC; van den Berg HM; van der Bom JC (Eds.) (2010): The RODIN study, research on determinants of inhibitors in patients with severe hemophilia A: a progress report. Abstract P4. Abstracts of the 3rd Annual Congress of the European Association for Haemophilia and Allied Disorders (Haemophilia, 16).

Gouw SC; van den Berg HM; van der Bom JC (Eds.) (2011): The RODIN study, research on determinants of inhibitors in patients with severe haemophilia A; towards the finish line. Abstract 21 (Haemophilia, 17).

Gringeri, A.; Monzini, M.; Tagariello, G.; Scaraggi, F. A.; Mannucci, P. M. (2006): Occurrence of inhibitors in previously untreated or minimally treated patients with haemophilia A after exposure to a plasma-derived solvent-detergent factor VIII concentrate. In *Haemophilia : the official journal of the World Federation of Hemophilia* 12 (2), pp. 128–132. DOI: 10.1111/j.1365-2516.2006.01201.x.

Gringeri A; Tagariello G; Mannucci PM (2000): A safety survey of inhibitor development on all of 130 italian users of the b-domain deleted recombinant factor VIII (BDDrFVIII) Refacto.

Gruppo R.; Chen H; Schroth P; Bray GL for the recombinate PUP study group (1998): Safety and immunogenicity of recombinant factor VIII (Recombinate) in previously untreated patients (PUPs): A 7.5 year update. Abstract 291.

Guérois, C.; Laurian, Y.; Rothschild, C.; Parquet-Gernez, A.; Duclos, A. M.; Négrier, C. et al. (1995): Incidence of factor VIII inhibitor development in severe hemophilia A patients treated only with one brand of highly purified plasma-derived concentrate. In *Thrombosis and haemostasis* 73 (2), pp. 215–218.

Halimeh, Susan; Bidlingmaier, Christoph; Heller, Christine; Gutsche, Sven; Holzhauer, Susanne; Kenet, Gili et al. (2013): Risk factors for high-titer inhibitor development in children with hemophilia A: results of a cohort study. In *BioMed research international* 2013, p. 901975. DOI: 10.1155/2013/901975.

Jansen M; Klukowska A; Komrska V; Laguna P (2013a): Low inhibitor incidence in previously untreated patients with severe haemophilia a treated with OCTANATE® – update from the PUP-GCP clinical trial. Poster PO102. In *Haemophilia : the official journal of the World Federation of Hemophilia* 19, pp. 10–82. DOI: 10.1111/hae.12083.

Jansen M; Klukowska A; Komrska V; Laguna P (2013b): Low inhibitor incidence in previously untreated patients with severe haemophilia A treated with octanate® - update from the PUP-GCP clinical trial. Abstract P2-84 1.

Jansen M; Klukowska A; Komrska V; Laguna P; Vdovin V (Eds.) (2013c): Low inhibitor incidence in previously untreated patients with severe haemophilia A treated with octanate – Update from the PUP-GCP clinical trial. Poster PB3.36-2. ABSTRACTS OF THE XXIV CONGRESS OF THE INTERNATIONAL SOCIETY ON THROMBOSIS AND HAEMOSTASIS, 29.06.-04.07. (Journal of Thrombosis and Haemostasis, 1 (suppl. 2)).

Klukowska, A.; Komrska, V.; Jansen, M.; Laguna, P. (2011): Low incidence of factor VIII inhibitors in previously untreated patients during prophylaxis, on-demand treatment and surgical procedures, with Octanate®. Interim report from an ongoing prospective clinical study. In *Haemophilia : the official journal of the World Federation of Hemophilia* 17 (3), pp. 399–406. DOI: 10.1111/j.1365-2516.2010.02428.x.

Klukowska, A.; Szczepański, T.; Vdovin, V.; Knaub, S.; Bichler, J.; Jansen, M. et al. (2018): Long-term tolerability, immunogenicity and efficacy of Nuwiq® (human-cl rhFVIII) in children with severe haemophilia A. In *Haemophilia : the official journal of the World Federation of Hemophilia* 24 (4), pp. 595–603. DOI: 10.1111/hae.13460.

Klukowska A; Jansen M; Komrska V; Laguna P; Vdovin V; Knaub S (2013): Latest Results From The PUP-GCP Clinical Trial: A Low Inhibitor Rate In Previously Untreated Patients With Severe Hemophilia A Treated With Octanate. In *Blood* (122(21)), p. 3596.

Klukowska A; Knaub S; Komrska V; Laguna P; Vdovin V; Jansen M (Eds.) (2014): Latest results from The PUP-GCP clinical trial: A low inhibitor rate in previously untreated patients with severe hemophilia A treated with Octanate (Haemophilia, 20 (Suppl. 3)).

Klukowska A; Laguna P; Belyanskaya L; Komrska V; Jansen M (Eds.) (2011): Octanate shows low inhibitor incidence in treatment of previously untreated patients with Haemophilia A. Abstract P01-3. 54. Jahrestagung Gesellschaft für Thrombose- und Hämostaseforschung (GTH). Wiesbaden, Germany, 16.-19.02. (Hämostaseologie, 1).

Klukowska A; Laguna P; Komrska V; Jansen M; Feddern J (Eds.) (2010): Low inhibitor incidence in previously untreated haemophilia A patients treated with Octanate®. Abstract P05-33. 54. Jahrestagung Gesellschaft für Thrombose- und Hämostaseforschung (GTH). Nürnberg, Germany, 24.-27. February (Hämostaseologie, 1).

Kreuz, W.; Gazengel, C.; Gonna, E.; Kellermann, E: and the European PUP/MTP Study Group (Eds.) (2001): 18 month experience with a sucrose-formulated Full-length rFactor VIII (rFVIII-SF) in previously untreated (PUPs) and minimally treated patients (MTPs) with severe Hemophilia A. Abstract A37. Annals of Hematology. V80 Suppl. 1.

Kreuz, Wolfhart; Ettingshausen, Carmen Escuriola; Zyschka, Alex; Oldenburg, Johannes; Saguer, Inmaculada Martinez; Ehrenforth, Silke; Klingebiel, Thomas (2002): Inhibitor development in previously untreated patients with hemophilia A. A prospective long-term follow-up comparing plasma-derived and recombinant products. In *Seminars in thrombosis and hemostasis* 28 (3), pp. 285–290. DOI: 10.1055/s-2002-32664.

Kreuz, Wolfhart; Gill, Joan C.; Rothschild, Chantal; Manco-Johnson, Marilyn J.; Lusher, Jeanne M.; Kellermann, Elke et al. (2005): Full-length sucrose-formulated recombinant factor VIII for treatment of previously untreated or minimally treated young children with severe haemophilia A. Results of an international clinical investigation. In *Thrombosis and haemostasis* 93 (3), pp. 457–467. DOI: 10.1160/TH03–10–0643.

Kreuz W; Auerswald GKH; Budde U; Lenk H (2004): Inhibitor incidence in previously untreated patients (PUP) with Hemophilia A and B - a 10-year-follow-up of the prospective multicenter study. In *Blood* (104 (11)), p. 3983.

Kurnik, K.; Bidlingmaier, C.; Engl, W.; Chehadeh, H.; Reipert, B.; Auerswald, G. (2010): New early prophylaxis regimen that avoids immunological danger signals can reduce FVIII inhibitor development. In *Haemophilia : the official journal of the World Federation of Hemophilia* 16 (2), pp. 256–262. DOI: 10.1111/j.1365-2516.2009.02122.x.

Kurnik K; Halimeh S; Manner D, Holzhauer S, Escuriola Ettingshausen C, Schobess E, Nowak-Gottl U (2009): Impact of treatment intensity and Factor VIII products on the development on high-titre inhibitors in children with severe hemophilia A: Results of a non-concurrent cohort study. Poster I-325. In *Blood* Vol 114, Issue 22.

Lusher, J.; Abildgaard, C.; Arkin, S.; Mannucci, P. M.; Zimmermann, R.; Schwartz, L.; Hurst, D. (2004): Human recombinant DNA-derived antihemophilic factor in the treatment of previously untreated patients with hemophilia A: final report on a hallmark clinical investigation. In *Journal of thrombosis and haemostasis : JTH* 2 (4), pp. 574–583. DOI: 10.1111/j.1538-7933.2004.00646.x.

Lusher, J. M. (1991): Viral safety and inhibitor development associated with monoclonal antibody-purified F VIII C. In *Annals of hematology* 63 (3), pp. 138–141.

Lusher, J. M.; Arkin, S.; Abildgaard, C. F.; Schwartz, R. S. (1993): Recombinant factor VIII for the treatment of previously untreated patients with hemophilia A. Safety, efficacy, and development of inhibitors. Kogenate Previously Untreated Patient Study Group. In *The New England journal of medicine* 328 (7), pp. 453–459. DOI: 10.1056/NEJM199302183280701.

Lusher, J. M.; Lee, C. A.; Kessler, C. M.; Bedrosian, C. L. (2003): The safety and efficacy of B-domain deleted recombinant factor VIII concentrate in patients with severe haemophilia A. In *Haemophilia : the official journal of the World Federation of Hemophilia* 9 (1), pp. 38–49.

Lusher, J. M.; Roth, D. A. (2005): The safety and efficacy of B-domain deleted recombinant factor VIII concentrates in patients with severe haemophilia A. An update. In *Haemophilia : the official journal of the World Federation of Hemophilia* 11 (3), pp. 292–293. DOI: 10.1111/j.1365-2516.2005.01099.x.

Lusher, J. M.; Salzman, P. M. (1990): Viral safety and inhibitor development associated with factor VIIIC ultra-purified from plasma in hemophiliacs previously unexposed to factor VIIIC concentrates. The Monoclate Study Group. In *Seminars in hematology* 27 (2 Suppl 2), pp. 1–7.

Lusher JM; Gringeri A; Hanno I; Rodriguez D (Eds.) (1999): Safety, efficacy and inhibitor development in previously untreated patients (PUPs) treated exclusively with recombinant b domain deleted recombinant FVIII (BDDrFVIII). Abstract 1037.

Luu, Huong; Spotts, Gerald; Gajek, Hartwig; Kriukov, Alex; Berg, Roger; Stephens, Darby et al. (2007): Safety Profile of Antihemophilic Factor (Recombinant), Plasma/Albumin-Free Method (rAHF-PFM) from Post-Authorization Safety Surveillance (PASS) Program. In *Blood* (110), p. 3972.

Maak B.; Nimtz-Talaska A.; Nowak-Göttl U;., Kentouche K., Schulze M., Wolf H., Schubert C., Halimeh S., Syrbe G., Eifrig B., Schuhen A.,Auerswald G.: (Eds.) (2012): Treatment of hemophilia A patients with FAKTOR VIII SDH INTERSERO: Interim report of a long-time post-marketing pharmacovigilance. Abstract No: P2-5. 56. Jahrestagung der Ges. für Thrombose-und Hämostaseforschung. St. Gallen, Schweiz.

Mancuso, M. E.; Mannucci, P. M.; Rocino, A.; Garagiola, I.; Tagliaferri, A.; Santagostino, E. (2012): Source and purity of factor VIII products as risk factors for inhibitor development in patients with hemophilia A. In *Journal of thrombosis and haemostasis : JTH* 10 (5), pp. 781–790. DOI: 10.1111/j.1538-7836.2012.04691.x.

Matysiak, M.; Bobrowska, H.; Balwierz, W.; Chybicka, A.; Kowalczyk, J. R.; Shaikh-Zaidi, R. et al. (2011): Clinical experience with Optivate®, high-purity factor VIII (FVIII) product with von Willebrand factor (VWF) in young children with haemophilia A. In *Haemophilia : the official journal of the World Federation of Hemophilia* 17 (5), pp. 737–742. DOI: 10.1111/j.1365-2516.2011.02600.x.

Mauser-Bunschoten, E. P.; Posthouwer, D.; Fischer, K.; van den Berg, H. M. (2007): Safety and efficacy of a plasma-derived monoclonal purified factor VIII concentrate during 10 years of follow-up. In *Haemophilia : the official journal of the World Federation of Hemophilia* 13 (6), pp. 697–700. DOI: 10.1111/j.1365-2516.2007.01554.x.

Mauser-Bunschoten, E. P.; van der Bom, J. G.; Bongers, M.; Twijnstra, M.; Roosendaal, G.; Fischer, K.; van den Berg, H. M. (2001): Purity of factor VIII product and incidence of inhibitors in previously untreated patients with haemophilia A. In *Haemophilia : the official journal of the World Federation of Hemophilia* 7 (4), pp. 364–368.

Musso, Roberto; Santagostino, Elena; Faradji, Albert; Iorio, Alfonso; van der Meer, Jan; Ingerslev, Jørgen et al. (2008): Safety and efficacy of sucrose-formulated full-length recombinant factor VIII. Experience in the standard clinical setting. In *Thrombosis and haemostasis* 99 (1), pp. 52–58. DOI: 10.1160/TH07–06–0409.

Oldenburg, J.; Goudemand, J.; Valentino, L.; Richards, M.; Luu, H.; Kriukov, A. et al. (2010): Postauthorization safety surveillance of ADVATE antihaemophilic factor (recombinant), plasma/albumin-free method demonstrates efficacy, safety and low-risk for immunogenicity in routine clinical practice. In *Haemophilia : the official journal of the World Federation of Hemophilia* 16 (6), pp. 866–877. DOI: 10.1111/j.1365-2516.2010.02332.x.

Peerlinck, K.; Rosendaal, F. R.; Vermylen, J. (1993): Incidence of inhibitor development in a group of young hemophilia A patients treated exclusively with lyophilized cryoprecipitate. In *Blood* 81 (12), pp. 3332–3335.

Peyvandi, Flora; Mannucci, Pier M.; Garagiola, Isabella; El-Beshlawy, Amal; Elalfy, Mohsen; Ramanan, Vijay et al. (2016): A Randomized Trial of Factor VIII and Neutralizing Antibodies in Hemophilia A. In *The New England journal of medicine* 374 (21), pp. 2054–2064. DOI: 10.1056/NEJMoa1516437.

Philipp CS; Shapiro AD; Gruppo RA; Bedrosian CL; Nissen S; Nguyen K (Eds.) (2001): Safety and Efficacy in previously untreated patients (PUPs) treated with recombinant b-domain deleted recombinant FVIII (BDDrFVIII). Abstract 157.

Pollmann, H.; Externest, D.; Ganser, A.; Eifrig, B.; Kreuz, W.; Lenk, H. et al. (2007): Efficacy, safety and tolerability of recombinant factor VIII (REFACTO) in patients with haemophilia A. Interim data from a postmarketing surveillance study in Germany and Austria. In *Haemophilia : the official journal of the World Federation of Hemophilia* 13 (2), pp. 131–143. DOI: 10.1111/j.1365-2516.2006.01416.x.

Pollmann, Hartmut; Klamroth, Robert; Vidovic, Natascha; Kriukov, Alexander Y.; Epstein, Joshua; Abraham, Ingo et al. (2013): Prophylaxis and quality of life in patients with hemophilia A during routine treatment with ADVATE antihemophilic factor (recombinant), plasma/albumin-free method in Germany. A subgroup analysis of the ADVATE PASS post-approval, non-interventional study. In *Annals of hematology* 92 (5), pp. 689–698. DOI: 10.1007/s00277-013-1678-4.

Pollmann H, Sabine H. (Ed.) (2001): Incidence of inhibitors in hemophilia A PUPs treated with ReFacto: First results from an pharmacovigilance evaluation in Germany. Abstract 2239.

Rothschild, C.; Gill, J.; Scharrer, I.; Bray, G. (2000): Transient inhibitors in the Recombinate PUP study. In *Thrombosis and haemostasis* 84 (1), pp. 145–146.

Rothschild, C.; Laurian, Y.; Satre, E. P.; Borel Derlon, A.; Chambost, H.; Moreau, P. et al. (1998): French previously untreated patients with severe hemophilia A after exposure to recombinant factor VIII. Incidence of inhibitor and evaluation of immune tolerance. In *Thrombosis and haemostasis* 80 (5), pp. 779–783.

Schwartz, R. S.; Abildgaard, C. F.; Aledort, L. M.; Arkin, S.; Bloom, A. L.; Brackmann, H. H. et al. (1990): Human recombinant DNA-derived antihemophilic factor (factor VIII) in the treatment of hemophilia A. recombinant Factor VIII Study Group. In *The New England journal of medicine* 323 (26), pp. 1800–1805. DOI: 10.1056/NEJM199012273232604.

Smith, M. P.; Giangrande, P.; Pollman, H.; Littlewood, R.; Kollmer, C.; Feingold, J. (2005): A postmarketing surveillance study of the safety and efficacy of ReFacto (St Louis-derived active substance) in patients with haemophilia A. In *Haemophilia : the official journal of the World Federation of Hemophilia* 11 (5), pp. 444–451. DOI: 10.1111/j.1365-2516.2005.01131.x.

Strauss, T.; Lubetsky, A.; Ravid, B.; Bashari, D.; Luboshitz, J.; Lalezari, S. et al. (2011): Recombinant factor concentrates may increase inhibitor development. A single centre cohort study. In *Haemophilia : the official journal of the World Federation of Hemophilia* 17 (4), pp. 625–629. DOI: 10.1111/j.1365-2516.2010.02464.x.

van den Berg, H. Marijke; Hashemi, S. Mojtaba; Fischer, Kathelijn; Petrini, Pia; Ljung, Rolf; Rafowicz, Anne et al. (2016): Increased inhibitor incidence in severe haemophilia A since 1990 attributable to more low titre inhibitors. In *Thrombosis and haemostasis* 115 (4), pp. 729–737. DOI: 10.1160/TH15-08-0692.

van der Bom, Johanna G.; Mauser-Bunschoten, Eveline P.; Fischer, Kathelijn; van den Berg, H. Marijke (2003): Age at first treatment and immune tolerance to factor VIII in severe hemophilia. In *Thrombosis and haemostasis* 89 (3), pp. 475–479.

Vepsäläinen, K.; Lassila, R.; Arola, M.; Huttunen, P.; Koskinen, S.; Ljung, R. et al. (2016): Inhibitor development in previously untreated patients with severe haemophilia A. A nationwide multicentre study in Finland. In *Haemophilia : the official journal of the World Federation of Hemophilia* 22 (5), pp. 721–729. DOI: 10.1111/hae.12974.

Vézina, C.; Carcao, M.; Infante-Rivard, C.; Lillicrap, D.; Stain, A. M.; Paradis, E. et al. (2014): Incidence and risk factors for inhibitor development in previously untreated severe haemophilia A patients born between 2005 and 2010. In *Haemophilia : the official journal of the World Federation of Hemophilia* 20 (6), pp. 771–776. DOI: 10.1111/hae.12479.

Yee, T. T.; Williams, M. D.; Hill, F. G.; Lee, C. A.; Pasi, K. J. (1997): Absence of inhibitors in previously untreated patients with severe haemophilia A after exposure to a single intermediate purity factor VIII product. In *Thrombosis and haemostasis* 78 (3), pp. 1027–1029.

Yoshioka, A.; Fukutake, K.; Takamatsu, J.; Shirahata, A. (2006): Clinical evaluation of recombinant factor VIII preparation (Kogenate) in previously treated patients with hemophilia A: descriptive meta-analysis of post-marketing study data. In *International journal of hematology* 84 (2), pp. 158–165. DOI: 10.1532/IJH97.06019.
