# Supplementary material for: Epidemiological Challenges in Rare Bleeding Disorders: FVIII Inhibitor Incidence in Haemophilia A Patients—A Known Issue of Unknown Origin
Source: Int J Environ Res Public Health. 2020 Dec 30;18(1):225. doi: 10.3390/ijerph18010225 (PMC7795862; doi:10.3390/ijerph18010225)
Supplement: Supplementary file 1 [file ijerph-18-00225-s001.zip › Supplement 5.docx]

| Brand name | Substance | Company | Recruiting started | (estimated) completion date | NCT number | Planned number of enrolled PUPs | Ongoing? | Inhibitors all [%] |
| --- | --- | --- | --- | --- | --- | --- | --- | --- |
| Kovaltry/Iblias^1^ | BAY81-8973 | Bayer | 06/2011 | 10/2020 | 01311648 | 50 | completed | -- |
| NovoEight* | Turoctocog alfa | NovoNordisk | 09/2012 | 12/2018 | 01493778 | 60 | completed | 43,1 |
| Nuviq/Vihuma* | Human-cl-rhFVIII | Octapharma | 02/2013 | 12/2019 | 01712438 | 110 | completed | 26,7 |
| Esperoct | turoctocog alfa pegol | NovoNordisk | 06/2014 | 11/2021 | 02137850 | 125 | yes | -- |
| Afstyla^1^ | rVIII‑SingleChain, (CSL627) | CSL Behring | 10/2014 | 01/2021 | 02172950 | 250 (PUPs + PTPs) | yes | -- |
| Elocta | rFVIIIFc | Bioverativ Therapeutics Inc. | 01/2015 | 06/2019 | 02234323 | 108 | completed | 31,1 |
| Adynovi | PEGylated Factor VIII (BAX 855) | Baxter | 12/2015 | 06/2023 | 02615691 | 120 | yes | -- |
| Voncento | pdFVIII | CSL Behring | No PUP study | | | | | |
| Jivi | Damoctocog alfa pegol | Bayer | No PUP study | | | | | |

FVIII products where PUP CTs were performed or discarded while/after the ClinGL came into effect. * = results published on clinicaltrials.gov, ^1^results are submitted to clinicaltrials.gov, but not yet published
